# Surgical Technique for Oral Mucosa Harvesting in Autologous Cultivated Oral Mucosal Epithelial Cell Transplantation for Ocular Surface Disorders

**DOI:** 10.7759/cureus.69648

**Published:** 2024-09-18

**Authors:** Hiroshi Toshida

**Affiliations:** 1 Ophthalmology, Juntendo University Shizuoka Hospital, Shizuoka, JPN

**Keywords:** ex vivo cultivated oral mucosal epithelial cell transplantation, limbal stem cell deficiency, ocural, oral mucosa, regenerative medicine

## Abstract

Ex vivo cultivated oral mucosal epithelial cell transplantation (COMET) was first introduced in Japan in June 2021. This technique is used to treat limbal stem cell deficiency (LSCD). This article provides a detailed description of one of the most critical steps in COMET, which is the harvesting of oral mucosa, along with accompanying videos. The samples harvested using this method were successfully cultured into cell sheets, which were then used in surgical procedures without complications.

## Introduction

Recent years have seen remarkable advances in medicine that have led to the introduction of new treatment technologies and options for intractable diseases for which adequate treatment was not previously available. One such advancement is ex vivo cultivated oral mucosal epithelial cell transplantation (COMET) [1]. COMET was first reported in 2004 by Nakamura et al. [2] as a method for transplanting a cultured sheet of oral mucosal epithelial cells to the ocular surface by using the amniotic membrane as part of the base in humans. The target disease of COMET is limbal stem cell deficiency (LSCD), a condition in which stem cells of the corneal epithelium disappear from the corneal limbus for a number of reasons, including congenital diseases such as aniridia, endogenous diseases such as Stevens-Johnson syndrome and ocular pemphigoid, idiopathic diseases of unknown etiology, trauma, and burns [1,3,4]. Such diseases and traumas can cause the conjunctival epithelium to invade the corneal surface, resulting in severe corneal opacification and, consequently, significant loss of vision. The primary candidates for COMET are patients with bilateral LSCD because unilateral LSCD is treated by human autologous corneal limbus-derived cultured corneal epithelial cell sheet transplantation, which was first described by Pellegrini et al. in 1997 [5].

The methods of transplanting a cultured cell sheet to treat LSCD are innovative and aimed at curing the disease. The treatment for unilateral LSCD, human autologous corneal limbus-derived cultured corneal epithelial cell sheet transplantation, involves the harvesting of a portion of healthy corneal limbal epithelial cells from the fellow, healthy eye and cultivation of the cells to create a sheet, which is then transplanted into the affected eye. The method uses a temperature-responsive culture dish, which allows for the sheet to be separated very cleanly from the culture container. Human autologous corneal limbus-derived cultured corneal epithelial cell sheet transplantation was the first such product to be commercialized and became available on the European market in 2015 under the name Holoclar® [6-8]. In Japan, a product for human autologous corneal limbus-derived cultured corneal epithelial cell sheet transplantation became available in 2020 and was marketed as Nepic® [9,10].

The first COMET product to become commercially available, developed by Nishida et al., was approved in Japan in 2021 and is sold under the name Ocural® [1,11]. This product also uses a temperature-responsive culture dish, which helps when removing the cell sheet from the dish.

Before COMET became commercially available, studies in many countries led to the publication of a number of clinical reports, each of which presented unique methods for COMET [1,3,4,11]. Most of these articles described postoperative results, the sheet itself, or the postoperative features of the ocular surface tissue; however, as far as we have investigated, there is only one previous report that details the methods used for oral mucosa harvesting [12]. Therefore, this report explains the details of the operative procedures required for Ocural® and includes videos taken while these procedures were performed on our patients after the product was marketed.

## Technical report

Ethical statement

The protocol for COMET treatment was reviewed and approved by the Ethics Committee of Juntendo University Shizuoka Hospital, Japan (approval no.: 886). The treated patients mentioned in this article provided written informed consent to undergo treatment, which complied with the guidelines for human studies and the World Medical Association Declaration of Helsinki. In addition, the patient shown in the videos provided written informed consent for inclusion of the videos in this article.

Patients indicated for treatment with Ocural®

According to Japanese guidelines [13], Ocural® is suitable for patients with one of two specific types of LSCD, as classified by Deng et al. [2]. The first type corresponds to stages IIB and III, involving limbal involvement exceeding 50% around the corneal limbus in the affected eye, with the affected area extending into the central 5-mm diameter zone that includes the central cornea. The second type corresponds to stage IIA, where the affected area also extends into the 5-mm diameter zone of the affected eye. This stage is identified after the removal of conjunctival scar tissue and, if necessary, following amniotic membrane transplantation, although these measures may be insufficient. It is important to note that these conditions also apply to Nepic®, which, as mentioned, is restricted to cases where the fellow eye is healthy.

Operative procedures for harvesting oral mucosa

Preparation Prior to Operation

Patients are advised to visit a dentist to treat any caries before scheduling the operation as the oral environment and smoking habits may impact culture results, which is recommended by the manufacturer. Additionally, the dental visit provides an opportunity to ensure that there are no scars or signs of inflammation on the oral mucosa and to confirm that there are suitable areas for tissue harvesting [13].

Immediately before the collection of oral mucosa, patients should clean the oral cavity and the area around the lips with toothpaste and iodine, ensuring to check for any history of iodine allergy.

Local Anesthesia

Two types of local anesthesia are administered to the patient. Lidocaine spray (Xylocaine® Pump Spray 8%, Sandoz Pharma K.K., Tokyo, Japan) is first applied to the operative field. This is followed by a submucosal injection of lidocaine containing epinephrine (Xylocaine® Injection 1％ with Epinephrine, Sandoz Pharma K.K., Tokyo, Japan). It is crucial to confirm any history of lidocaine allergy before proceeding with these anesthetic procedures.

Harvesting of Oral Mucosal Tissue

**Figure 1:**
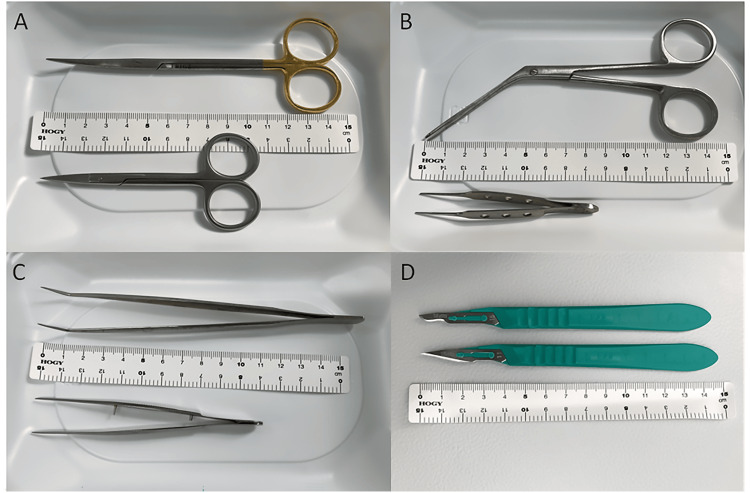
Images of surgical instruments prepared for this surgery The top row of photos A through C shows the newly purchased instruments for this surgery, while the bottom row shows the instruments typically used in standard ophthalmic surgeries. Photo D shows scalpels, with No. 15 used in this surgery in the top row and the No. 11 prepared as a backup in the bottom row. The names of the instruments in the top row are listed sequentially. A: T/C Metzenbaum-Fino Scissors; B: ENT Foreign Body Forceps; C: Curved, Fine-Tipped, Non-Toothed Tweezers; D: Kai Industries Scalpel No. 15

Oral mucosal tissue is harvested at a depth that includes the basal layer, which contains epithelial stem cells. Instruments such as scissors, forceps, and a scalpel, which were newly purchased and longer than those typically used in standard ophthalmic surgeries, are used for this procedure. A list of these special instruments and images is provided in Table 1 and Figure 1. The dimensions for the excision are specified in the product manual as a 10 x 5 mm rectangle. However, a boat-shaped incision is made instead to obtain a specimen measuring 17 x 5 mm using a No. 15 scalpel (Kai Industries, Gifu, Japan) (Video 1).

**Table 1:** A list of surgical instruments prepared for this surgery T/C: Tungsten Carbide; ENT: ear, nose, and throat

Surgical instruments	Distributor	Model number	Images in Figure 1
T/C Metzenbaum-Fino Scissors, 14.5 cm, curved, sharp/sharp type	Nippon Fritz Medico Co., Ltd., Tokyo, Japan	B023-014E	A
ENT Foreign Body Forceps (General Examination) Barley	U-MED Industrial Inc., Tokyo, Japan	SM-22353	B
Curved, Fine-Tipped, Non-Toothed Tweezers, 160 mm	AS ONE Co., Ltd., Osaka, Japan	No. 4	C
Kai Industries Scalpel, No. 15	Kai Industries, Gifu, Japan	515-A	D
Disposable Mouth Gag NaviMouth L-Type	NAVIS (AS ONE) Co., Ltd., Osaka, Japan	7-4323-03	none (see Video 1)

**Video 1:** Harvesting of oral mucosa for Ocural® (case 1) After spraying lidocaine and disinfecting around the mouth with iodine solution, a mouth gag was inserted, and the area of the oral mucosa to be excised was marked. During this process, the ink bled when the marking pen was applied directly to the oral mucosa. Local anesthesia was then administered, and the oral mucosa was incised with a scalpel, carefully following the marked area. The harvested tissue was immediately placed in a container. The incision site on the oral mucosa was sutured with silk thread while controlling bleeding.

Markings were made on the oral mucosa prior to incision. In the first case, an attempt was made to mark the oral mucosa directly with a marker pen, but the ink bled (Video 1). In the second case, following the previous report [12], we applied ink only to the edges of a surgical paper with memory markers cut into a boat shape and placed it on the oral mucosa, which allowed us to draw clean lines. In the second case, we further improved the method of tissue collection by making an incision in the surface layer of the oral mucosa and then hooking a 6-0 nylon thread to one side and pulling on it when removing the specimen (Video 2).

**Video 2:** Improved method for harvesting technique of oral mucosa for Ocural® (case 2) In case 2, to address the issue of ink bleeding during the marking of the oral mucosa in case 1, a piece of paper cut to the planned incision size was prepared, and ink was applied only to the edges. This allowed for clean marking on the oral mucosa. Additionally, a thread was hooked onto the tip of the incised oral mucosa to lift it more easily during excision. The oral mucosa was then carefully harvested.

The tissue is collected with long forceps and scissors, immediately immersed in physiological saline with antibacterial drugs, washed with diluted iodine solution, stored in a transport tube in an ice box, and then transferred to Japan Tissue Engineering Co., Ltd. in Aichi, Japan for enzymatic treatment and subculturing on 3T3 cell-derived feeder cells.

Wound Care

The wound is closed with three to four stitches of 6-0 silk after performing compression hemostasis with gauze and electrocoagulation. Although suture removal is sometimes deemed unnecessary as they tend to fall off naturally, stitches were removed on postoperative day 7 to ensure proper healing.

Cultivated oral mucosal epithelial cell transplantation

After confirming an adequate growth of epithelial cells over two weeks, the transplantation is scheduled four weeks from the start of the culture. On the surgery day, the cell sheets are transported to the operating facility, where under local anesthesia, the overgrown bulbar conjunctiva is removed and the sheet transplanted. Finally, fluorescein dye checks for epithelial defects and a therapeutic contact lens is placed for protection (Video 3). The postoperative course was favorable, and the short-term treatment outcomes were as previously reported [11].

**Video 3:** Digest video on cultivated oral mucosal epithelial cell transplantation In this video, we transplanted cultivated oral mucosal epithelial cells that had been harvested in Video 2. This Video 3 is a simplified digest version.

## Discussion

Ocural®, the world’s first commercially available COMET product, consists of a tissue transport set, which includes tubes for carrying oral mucosal tissue and storage blood harvested at a medical institution, and a cultured oral mucosal epithelium package [10,11]. The biggest advantage of this product is that cell culture operations are performed by specialized vendors. Another point worth noting is that this product uses a temperature-responsive culture dish, which is advantageous because it allows for the cell sheet to be peeled off when a suitable temperature is reached [9,14].

One of the concerns when harvesting oral mucosa samples is the possibility of contamination by pathogens such as oral bacteria, fungi, and mycoplasma. To prevent such contamination, specimens are harvested after preoperative dental caries treatment, and dental brushing and oral disinfection are performed immediately before the operation. In all cases where the method for harvesting oral mucosa was demonstrated in the accompanying videos, the cell cultures were successful, resulting in cell sheets that were used in COMET surgeries without any complications. In a previous report, we examined the characteristics of anterior segment specimens obtained from cases that underwent superficial keratoplasty six months postoperatively, and it was shown that the oral mucosa-derived cells exhibited specific features. Although cell culture proceeded smoothly even in Case 1, shown in Video 1, where the marker ink bled onto the oral mucosa, it should be noted that some studies have pointed out the potential toxicity of markers. Therefore, it is preferable to use a method with less ink application during marking, as demonstrated in Video 2.

In all cases where the method for harvesting oral mucosa was demonstrated in the accompanying videos, the cell cultures were successful, resulting in cell sheets that were safely used in COMET surgeries. In a previous report, we examined the characteristics of anterior segment specimens obtained from cases that underwent superficial keratoplasty six months postoperatively, and it was shown that the oral mucosa-derived cells exhibited specific features. Even in Case 1, shown in Video 1, where the marker ink bled onto the oral mucosa, the cell culture proceeded smoothly. However, it should be noted that the dye used in the marker, Hexamethyl pararosaniline chloride (known as Crystal Violet or Gentian Violet), has been reported in the literature to have toxic effects on vascular cells and corneal endothelium [15,16]. Therefore, as demonstrated in Video 2, it is preferable to minimize the amount of ink applied during marking.

As for the methods for harvesting oral mucosa samples, reports have already been published on harvesting methods and their use in oral surgery and other fields [12]. For Ocural®, harvesting of oral mucosa involves making an incision with a scalpel and harvesting the specimen from the buccal mucosa; this method was used in all cases at the clinical trial stage and is therefore recommended for use in clinical practice. We changed the shape of the excision from a rectangular shape measuring 10 x 5 mm, as described in the Ocural® manual, to a boat shape measuring 17.5 x 5 mm. Despite these changes, our method obtains a satisfactory number of basal cells (the number obtained when a rectangular shape is used has not been disclosed by the company).

In a previous report that described the biopsy size, the smallest and largest areas of the excised oral mucosa were 2 mm^2^ and 200 mm^2^, respectively [12]. In addition to harvesting by scalpel excision, as in the case of Ocural®, others have reported the use of a biopsy punch. This method has been recommended because oral mucosa was harvested by biopsy punch in clinical trials. Biopsy punches with a diameter of 3, 6, and 8 mm were used, corresponding to areas of 4.71 mm^2^, 9.42 mm^2^, and 50.24 mm^2^, respectively. According to the Ocural®‘s manual, the area is 50 mm^2 ^(because a 10 x 5 mm rectangle should be excised), which is equivalent to the area obtained with the 8-mm diameter biopsy punch. However, a study that compared cell densities when using scalpel excision harvesting versus a biopsy punch reported that the former had a higher cell density. In cases where there is a concern that the number of cells may be insufficient, it seems feasible to harvest biopsy punch samples at multiple sites. Given that the biopsy punch method does not require suturing, it is highly likely to become popular, with more evidence accumulating in the future. Although most reports that specified the site of oral mucosa harvest described the use of the buccal area, the labial mucosa may be an easier site, and hence, a more convenient method might be established in the future as evidence accumulates.

As of August 2024, three types of regenerative medicine products are commercially available in Japan for the treatment of LSCD: Ocural®, Nepic®, and Sakracy®. Sakracy® is a human oral mucosa-derived epithelial cell sheet that uses a human amniotic membrane substrate [2,17]. Ocural® and Sakracy® are COMET products, which are used when both eyes are affected, and Nepic® is a human corneal limbus-derived corneal epithelial cell sheet for LSCD patients with healthy fellow eyes, as described above. Ocural® is indicated if there is no symblepharon formation, and Sakracy® is indicated if symblepharon is present [11]. The Sakracy® manual recommends the use of a biopsy punch for harvesting oral mucosa. With all three products, a cultured sheet is created in a similar manner by harvesting oral mucosa and transplanting cultured cells into the affected eye.

In Japan, the Ocural® guidelines [13] include some unique criteria for operating physicians. Based on the idea that physicians performing the procedures should be required to have knowledge and experience working as an ophthalmologist and experience in performing corneal transplantation surgery, the guidelines specify that physicians must meet all of the following criteria: (1) be a member of the Japan Cornea Society and the Keratoplasty Society of Japan and be a specialist certified by the Japanese Ophthalmological Society; (2) have experience in performing corneal transplantation surgery in more than five patients; and (3) have completed training in courses organized by manufacturers/sellers. The harvesting of oral mucosal tissue has been authorized only for physicians, excluding dentists. When physicians other than ophthalmology specialists who meet the above criteria perform harvesting of oral mucosal tissues, they are required to complete training in tissue harvesting at a workshop organized by the manufacturer. This situation is a result of the restrictions imposed by Japan's specific health insurance system, and as a result, ophthalmology specialists are currently the ones performing the harvesting of oral mucosal tissue in Japan. However, since oral surgeons, dentists, and otolaryngologists are more familiar with the techniques and procedures within the oral cavity, it would be more practical to have them perform the harvest of oral mucosa in countries without such restrictions.

Safe harvest of oral mucosa samples is an important issue that affects postoperative outcomes. Even if a skilled physician who fulfills the above criteria, including criterion (3), harvests the oral mucosa and follows the conventional protocol and no contamination results from the procedure, there is still a possibility that the cultured cells do not develop correctly or that the patient cannot be hospitalized or undergo surgery on the scheduled day of surgery because they have developed other diseases or had an accident. The success or failure of COMET is evident in the condition of the ocular surface and improvement in postoperative visual acuity. With respect to postoperative outcomes, on the basis of cases they have experienced in the past, the authors believe that anti-inflammatory treatment and early resolution of epithelial erosion are particularly important [11]. In addition to these postoperative management measures, operative techniques appear to be very important for the success of the operation.

## Conclusions

In the treatment of LSCD using COMET, precision in each step, from mucosal harvesting to cell culture and sheet transplantation, is crucial for success. This report demonstrates the procedures for oral mucosa tissue harvesting, which is the very first step in the Ocural® protocol, through video. For physicians and healthcare professionals, particularly ophthalmologists, who are performing this procedure for the first time, enhancing the precision of these complex techniques is essential to improving the success rate of LSCD treatment.
